# The Concurrent Association of Magnesium and Calcium Deficiencies with Cognitive Function in Older Hospitalized Adults

**DOI:** 10.3390/nu16213756

**Published:** 2024-10-31

**Authors:** Ganna Kravchenko, Serena S. Stephenson, Agnieszka Gutowska, Karolina Klimek, Zuzanna Chrząstek, Małgorzata Pigłowska, Tomasz Kostka, Bartłomiej K. Sołtysik

**Affiliations:** Department of Geriatrics, Healthy Ageing Research Centre (HARC), Central Teaching Hospital of the Medical University of Lodz, Pomorska 251, 92-213 Łódź, Poland

**Keywords:** hypomagnesemia, hypocalcemia, cognitive decline, seniors

## Abstract

**Background/Objectives**: Hypomagnesemia and hypocalcemia are common conditions among older adults that may contribute to cognitive decline. However, most of the existing research has focused primarily on dietary intake rather than the actual serum levels of these nutrients or examined them separately. This study aims to investigate the relationship between hypomagnesemia, hypocalcemia, and the concurrent presence of both deficiencies in relation to cognitive performance among seniors. **Methods**: A total of 1220 hospitalized patients aged 60 and older were included in the analysis. The participants were categorized into four groups: those with normal serum levels of magnesium and calcium, those with hypomagnesemia, those with hypocalcemia, and those with both serum magnesium and calcium deficiencies. To evaluate the potential influence of age, sex, common comorbidities, and disturbances in magnesium and calcium levels on cognitive performance, two general linear models were employed, using the Mini-Mental State Examination (MMSE) and Clock-Drawing Test (CDT) as dependent variables. **Results**: After adjusting for age, sex, body mass index, and comorbidities, the mean values for the MMSE and CDT were 23.33 (95%CI: 22.89–23.79) and 5.56 (95%CI: 5.29–5.83) for the group with normomagnesemia and normocalcemia, 22.59 (95%CI: 21.94–23.24) and 5.16 (95%CI: 4.77–5.54) for the group with hypomagnesemia, 19.53 (95%CI: 18.36–20.70) and 4.52 (95%CI: 3.83–5.21) for the group with hypocalcemia, and 21.14 (95%CI 19.99–22.29) and 4.28 (95%CI 3.61–4.95) for the group with both hypomagnesemia and hypocalcemia, respectively. Magnesium and calcium deficiencies contributed to MMSE and CDT variance in the general linear models. **Conclusions**: Our findings indicate that in addition to age, body mass index, and chronic heart failure, both hypomagnesemia and hypocalcemia are associated with reduced cognitive performance.

## 1. Introduction

Aging is a complex process characterized by multiple pathophysiological changes that significantly impact cognitive function. Hypomagnesemia and hypocalcemia are common in seniors and exacerbate cognitive decline [1,2]. These conditions are often interrelated, and their prevalence and impact on cognitive deterioration have gained increasing attention in geriatric research. Magnesium (Mg) deficiency hinders energy production, which is vital for mitochondrial ATP production, and reduces the antioxidative capacity essential for aging organisms’ defense against free radical damage. Mg functions as an antioxidant, protecting mitochondria from reactive oxygen species. Chronic inflammation and oxidative stress are essential components of various age-related illnesses. Prolonged Mg insufficiency leads to increased free radical generation and mild inflammation [3,4]. Mg acts as a positive regulator of synaptic plasticity. In vitro studies show that increasing the Mg^2+^ concentration within the physiological range in the extracellular fluid leads to a sustained enhancement of synaptic plasticity in cultured hippocampal neuron networks [5]. Furthermore, this ion modulates N-methyl-D-aspartate (NMDA) receptors, which are crucial for synaptic transmission, and stabilizes neuronal cell membranes [6]. Due to these crucial roles within the nervous system, Mg is of intense interest for the potential prevention and treatment of neurological disorders [7,8], especially given the frequent association between aging and hypomagnesemia [3,9,10].

Calcium-dependent signals are primary activators of molecular processes supporting learning and memory. Disruption in calcium (Ca) levels within the aging brain is suggested to be a fundamental factor in the cognitive deterioration associated with aging [9]. Ca’s role in neurotransmission and neuronal excitability is well documented, and its dysregulation is linked to cognitive deficits and neurodegenerative diseases [4]. Aging is characterized by a decline in the body’s ability to maintain homeostasis, including in electrolyte balance. The prevalence of hypomagnesemia and hypocalcemia increases with age due to factors such as reduced dietary intake, impaired absorption, and chronic diseases [11]. These ion disturbances may exacerbate cognitive decline, leading to conditions such as mild cognitive impairment (MCI) and dementia [4]. Data analysis from participants aged 60 years and older in the National Health and Nutrition Examination Survey (NHANES), USA, indicated that higher Mg intake is independently linked to better cognitive performance [12]. Regarding Ca, the data are more controversial—some studies have shown a decreased risk of dementia [1], while others have found opposite results [13,14]. 

A 17-year prospective cohort study involving 1081 older adults aged 60 and above found that increased self-reported dietary intakes of Ca and Mg were associated with a reduced risk of all-cause dementia [1]. Another study examined the effects of dietary Ca and Mg intake and the Ca:Mg ratio in an older population over a 5-year follow-up period. The findings revealed that participants with the lowest levels of dietary Ca and Mg had the highest incidence of dementia [15]. One cross-sectional research study conducted in 94 older subjects revealed that serum Ca and Ma levels are associated with better cognitive performance [16]. Similar work performed in younger participants obtained comparable results [17].

Most studies have focused on dietary intake rather than the actual serum levels of these nutrients. To the best of our knowledge, no existing studies have assessed concurrently the relationship between serum Mg and Ca concentrations and cognitive function in advanced age patients. This research aims to provide insights on how Ca and Mg metabolism may relate to cognitive functioning in seniors. Therefore, the fundamental objective of this study is to determine whether, and how, hypomagnesemia, hypocalcemia, and factors such as sex, age, body mass index (BMI), and comorbidities affect cognitive functions in the older hospitalized population.

## 2. Materials and Methods

### 2.1. Patients

For this cross-sectional study, patients were recruited from the Central Veterans Hospital in Lodz, Poland. The inclusion criteria were senior adults aged 60 years old and more hospitalized at the Department of Geriatrics who were able to utilize efficient verbal communication as well as to give informed consent. Considering rehospitalizations, consecutive admissions of the same subjects over the years were excluded from this analysis. After screening, 1220 patients (876 women and 344 men), consecutively evaluated with complete data, met the inclusion criteria and were enrolled in the analysis.

### 2.2. Anamnesis

Upon admission, a detailed examination of the patient’s medical history was performed. The presence of arterial hypertension (HT), diabetes mellitus type 2 (DM), lipid disorders, current or prior stroke, coronary artery disease (CAD), current or prior myocardial infarction (MI), atrial fibrillation (AF), chronic heart failure (HF), and chronic kidney disease (CKD), as evaluated by the BIS1 formula [18], was scrutinized.

### 2.3. Measurements

The BMI was calculated by dividing the weight (in kilograms) by the height squared (in meters). Serum Mg and Ca concentrations were measured for every participant in mmol/L. Magnesium was established using the colorimetric method with xylidyl blue, whereas calcium was determined with Arsenazo III and a Beckman Coulter Dx700 AU analyzer (Brea, CA, USA). Hypomagnesemia was defined as a serum level of Mg lower than 0.77 mmol/L, while hypocalcemia was defined as a concentration of Ca below 2.2 mmol/L [19]. Then, the Ca:Mg ratio was calculated [20]. The laboratory parameters were obtained upon the patients’ admission. In the present study, the Mini-Mental State Examination (MMSE) [21] and Clock-Drawing Test (CDT) [14] were performed for every patient by the departmental psychologist. The sum of the scores from the MMSE obtained for each of them provides a total score ranging from 0 to 30. Scores from 26 to 24 were defined as mild cognitive impairment (MCI), and results of 23 points and below were defined as dementia. CDT was assessed by the Sunderland scoring system (from 1 to 10 points), with higher numbers indicating better performance [22,23].

### 2.4. Statistical Analysis

The normality of distribution was verified with the help of the Shapiro–Wilk test. As several variables were not normally distributed, the data have been presented both as the mean ± standard deviation and the median (interquartile differences from the first (25%) quartile to the third (75%) quartile). Qualitative variables, such as sex and the presence of disease, were presented as raw numbers and percentages for the group. Patients were divided into four groups: (1) those with normal levels of Mg and Ca; (2) those with hypomagnesemia; (3) those with hypocalcemia; and (4) those with both serum Mg and Ca deficits. Groups were compared using the Kruskal–Wallis test (for quantitative variables) and the chi-square test (for qualitative variables). Pairwise comparisons between the average ranks of the 4 groups were performed using the Bonferroni procedure. Ca and Mg were also compared after dichotomization of the participants according to 24 points for the MMSE and 5 points for the CDT cut-off values, and the cognitive test scores were compared between the Ca and Mg quartile values.

To assess the potential influence of age, sex, common concomitant diseases, and ion disturbances on cognitive performance, two general linear models with the MMSE and CDT as the dependent variables were constructed, and a multiple comparison using Fisher’s least-significant difference procedure was performed to determine which means are significantly different. As the values of cognitive tests were not normally distributed, logarithmization was performed, and the obtained values were reapplied in the general linear models. Statistical significance was set at *p* ≤ 0.05. Statistical analysis was performed using Statistica 13.1, Statsoft, Poland.

### 2.5. Ethical Considerations

The study was conducted according to the guidelines of the Declaration of Helsinki and approved by the Ethics Committee of the Medical University of Lodz (approval number: RNN/68/23/KE; 18 April 2023).

## 3. Results

Table 1 presents the basic data categorized into groups based on the Mg and Ca serum levels: normomagnesemia with normocalcemia (Group I), hypomagnesemia with normocalcemia (Group II), normomagnesemia with hypocalcemia (Group III), and hypomagnesemia with hypocalcemia (Group IV). Subjects in Group III were significantly older than those in Groups I and II. Participants in Groups III and IV exhibited lower BMI results compared with Group II. Group III and IV subjects demonstrated poorer outcomes on the MMSE and CDT than Groups I and II. Subjects in Group II took significantly more medications in comparison with Group I, while Group III took significantly less medications in comparison with Groups I, II and IV. Regarding concomitant conditions, Group II subjects had the highest prevalence of type 2 diabetes, stroke, and CAD. Subjects in Group I had the highest prevalence of lipid disorders but the lowest prevalence of AF and HF.

Subsequently, in Table 2, the cognitive test scores were compared according to the presence or absence of specific concomitant conditions. Conditions such as stroke and HF were associated with lower MMSE scores. The CDT, AF, HF, and CKD were related with lower test results. In contrast, the presence of HT and lipid disorders was associated with better cognitive performance in the MMSE; however, subjects with arterial hypertension were significantly younger (z = 3.84; *p* < 0.001). The same results were found in the CDT for participants with lipid disorders with a similar age difference—higher lipid disorder prevalence was associated with a younger age (z = 6.81; *p* < 0.001).

In our population, calcium (Ca) showed a positive correlation with both the MMSE and the CDT (rho = 0.17, *p* = 0.001; rho = 0.13, *p* = 0.001, respectively). Similarly, magnesium (Mg) had a positive correlation with the MMSE and CDT (rho = 0.06, *p* = 0.02; rho = 0.06, *p* = 0.02). Additionally, Ca was positively correlated with the BMI, while no correlation was observed between Mg and the BMI (rho = 0.11, *p* = 0.001; rho = 0.01, *p* = 0.6).

Participants with MMSE scores below 24 (indicating dementia; *n* = 558) showed significantly lower Ca and Mg concentrations compared with the normal MMSE group. Subjects with CDT scores below 5 (*n* = 524) had significantly lower Ca concentrations. MMSE and CDT scores generally increased with quartiles of the Ca and Mg concentrations, with significant differences between Ca Quartile 1 and Quartiles 2, 3, and 4 for the MMSE, between Ca Quartile 1 and Quartiles 3 and 4 for the CDT, and significant differences between Mg Quartile 1 and Quartile 4 for both the MMSE and the CDT.

**Figure 1:**
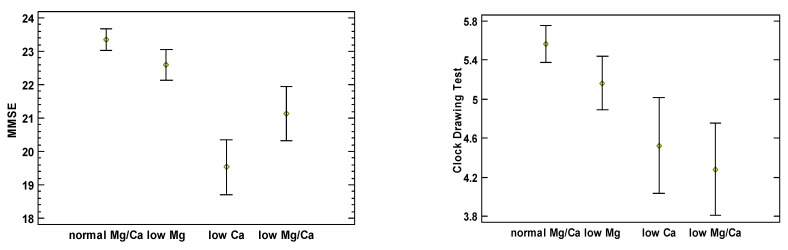
Comparison of MMSE and CDT scores between the four tested groups, adjusted for age, sex, BMI, and comorbidities.

Figure 1 illustrates each study cohort’s mean and 95% confidence intervals of MMSE and CDT scores adjusted for age, sex, BMI, and comorbidities. It shows generally inverse associations between the test scores and the presence of Mg and Ca deficiencies. MMSE and CDT scores for the group with normomagnesemia and normocalcemia were 23.33 (95%CI: 22.89–23.79) and 5.56 (95%CI: 5.29–5.83), 22.59 (95%CI: 21.94–23.24) and 5.16 (95%CI: 4.77–5.54) for the group with hypomagnesemia, 19.53 (95%CI: 18.36–20.70) and 4.52 (95%CI: 3.83–5.21) for the group with hypocalcemia, and 21.14 (95%CI 19.99–22.29) and 4.28 (95%CI 3.61–4.95) for the group with both hypomagnesemia and hypocalcemia, respectively. Groups III and IV had lower MMSE scores compared with Groups I and II. Group IV had lower CDT scores compared with Groups I and II, while group III had lower CDT scores compared with group I. Additionally, we calculated the Ca to Mg ratio; however, neither the MMSE nor the CDT scores expressed any correlation with this variable.

In the final analysis, we employed generalized linear models for the MMSE and CDT variables, as shown in Table 3. Due to the absence of a normal distribution and variance homogeneity, the variables were logarithmized; however, this did not affect the influence of the independent variables on the dependent ones.

Age has a noticeable negative effect on MMSE scores, with cognitive performance decreasing by 0.21 points for each additional year. Moreover, a higher BMI is linked to better MMSE results, with each unit increase in the BMI contributing to a 0.18-point rise in scores. The most significant impact comes from low calcium levels, with MMSE scores lower by 3.81 points compared with those for the normal Mg/Ca group.

For the CDT, age similarly reduces the scores by 0.13 points per year, indicating a gradual cognitive decline. A higher BMI slightly improves CDT scores, with a 0.09-point increase for each additional BMI unit. The combination of low magnesium and calcium leads to the greatest reduction in CDT performance, lowering scores by 1.28 points when compared with the normal Mg/Ca group.

## 4. Discussion

To the best of our knowledge, this study is the first to investigate the concurrent disturbances in serum Mg and Ca concentrations among older adults and their relationship with cognitive impairments. Our findings suggest that along with age, BMI, and HF, both hypomagnesemia and hypocalcemia are associated with reduced cognitive performance. This consistent structure was observed across two independent cognitive assessment tools used in the geriatric population.

The importance of Mg ions in the central nervous system is well established. Mg deficiency in the brain disrupts various cognitive functions, including maintaining the stability and integrity of cell membranes, regulating the reactivity of N-methyl-D-aspartate (NMDA) receptors to external stimuli, and modulating Ca levels [6]. Furthermore, Mg serves a pivotal role in detoxification processes, inhibits neuroinflammation, reduces beta-amyloid production [6,24], and prevents the phosphorylation of Tau protein [3]. Some clinical studies advocate that a diet high in Mg may reduce the risk of vascular and other types of dementia [3]. Additional research indicates that high-Mg supplementation can independently enhance cognitive functions in older adults [12,25,26,27], including preventing memory loss. For instance, some studies found that hypomagnesemia was linked to significantly slower reaction times [28] or attention and executive and language abilities in adults [8]. This research indicates that inadequate serum Mg levels in the geriatric population may be associated with poorer mental performance.

However, other studies offer a more nuanced perspective. A multicenter study involving hemodialysis patients revealed a U-shaped relationship between serum Mg levels and MCI, with both Mg deficiency and excess being linked to worse cognitive outcomes [29]. Similarly, a study conducted in the Rotterdam population found that both low and high Mg concentrations were associated with a higher risk of dementia [30]. Another longitudinal study, which tracked over 2500 participants for 25 years, found that hypomagnesemia was associated with an increased risk of dementia, without highlighting a specific cognitive domain as being predominantly affected [31]. A systematic review of serum Mg’s effects on Alzheimer’s disease (AD) suggests a correlation between lower Mg levels and AD, although the evidence is limited [32]. Notably, significantly increased Mg levels have been linked to greater brain volume and a reduced risk of cerebrovascular issues, suggesting that Mg may play a crucial role in neurodegeneration and cerebrovascular disease [33]. This research implies that Mg might not be directly associated with AD but could be more relevant in the context of neurodegeneration and vascular dementia.

In our study, after adjusting for age, sex, BMI, and comorbidities, participants with hypomagnesemia scored 0.93 points lower on the MMSE when compared with the normal Mg/Ca group. Although the difference in the CDT scores was 0.27 points lower, it was not statistically significant. Our findings align with the current literature, which suggests a possible link between reduced Mg levels and impaired cognitive performance. However, this relationship could be bidirectional: while hypomagnesemia may lead to poorer cognitive performance, impaired cognitive function could also contribute to lower Mg levels due to nutritional deficiencies.

Ca metabolism disorders are more complex than those of Mg. Ca plays numerous roles in the nervous system, including in the calcium-dependent regulation of transcription factors involved in memory formation [4]. Neuronal Ca homeostasis is crucial in the pathogenesis of AD by promoting beta-amyloid accumulation and neurofibrillary tangle formation [34,35,36]. Those mechanisms were demonstrated in vitro and in vivo in different cell models [37,38]. Some studies have reported positive effects from agents that modulate Ca channel activity, such as memantine [39,40] and nimodipine [41,42].

Regarding serum Ca concentrations, some research studies indicate a link between hypercalcemia and an increased risk of AD [43,44]. Conversely, other studies suggest poorer cognitive performance and hypocalcemia in the context of hypoparathyroidism [45]. One study found that individuals with AD had significantly lower serum levels of Ca and phosphorus, suggesting that this relationship may not be solely due to aging but also be linked to the pathology of age-dependent disorders [46]. Another study proposed that elevated serum Ca levels might reduce the risk of AD [47]. Additionally, lower serum Ca has been potentially associated with an increased risk of converting from MCI to AD [48]. A deficiency in vitamin D3 has also been suggested as a potential cause of dementia [49].

Our findings align with the literature on the cognition–calcium axis. Subjects with hypocalcemia presented worse cognitive test results in both the MMSE and the CDT. The impact of hypercalcemia on cognitive functions, such as in the case of hyperparathyroidism, is well documented. Increased extracellular Ca leads to greater cytosolic influx and cognitive deterioration. However, both our study and some cited studies indicate that a similar relationship may exist between hypocalcemia and cognitive function. Hypocalcemia can result from a poor diet, reduced intestinal Ca absorption, and vitamin D3 deficiency, which may be directly related to cognitive disorders. It is essential to determine whether poorer cognitive performance generates hypocalcemia, whether hypocalcemia causes worse cognitive performance, or whether the abovementioned relationship depends on other undetermined factors.

The idea of testing both serum Mg and Ca and cognitive functions remains largely unexplored. The Shanghai Aging Study measured dietary Mg and Ca intake and assessed their association with cognitive impairment. It found that high Mg intake with low Ca intake was associated with an elevated risk of dementia. Furthermore, subjects with the lowest intake of both Mg and Ca had the highest rates of dementia. However, this study refers to the dietary intake but not the blood concentration of these ions [15]. Similar conclusions were drawn by Ozawa et al. in a Japanese study, which found that a diet rich in potassium, Ca, and Mg reduced the all-cause dementia risk, with an emphasis on vascular dementia [1]. In our study, subjects with hypomagnesemia and hypocalcemia had significantly worse MMSE results compared with subjects with normal concentrations of both Mg and Ca. In the CDT, only combined hypomagnesemia and hypocalcemia was associated with worse cognitive performance. These results suggest the need for a broader perspective. In multivariate models, the relationship between BMI and cognitive test results, like that for age, is noteworthy. In both tests, a lower BMI is associated with poorer cognitive performance. This relationship is well documented [50,51,52], with a malnutrition prevalence of 32.5% in demented people [53]. Some studies suggest that oral dysfunction [54], vitamin deficiencies [55,56,57], or specific diets [58,59] may promote the incidence of dementia. The above facts, along with our study results, prompt consideration of whether deficiencies in macronutrients such as Ca and Mg and dementia are both a cause and consequence.

Interestingly, our results showed that patients with arterial hypertension and lipid disorders had higher scores in the MMSE and the CDT. Our finding aligns with a prospective research study on patients aged 85 years and older in Finland [60]. The authors demonstrated that a history of hypertension was associated with a lower probability of dementia in a 9-year follow-up period. This relationship may be explained by the J-curve phenomenon, where cognitive impairments are more common in individuals with low blood pressure than those with controlled high blood pressure [61,62], or because of the significantly lower age of subjects with arterial hypertension. Although lipid disorders and HT in middle age are proven risk factors for developing dementia in older life [63], some studies have shown that cholesterol levels tend to decrease with age, and subjects with AD present lower levels of serum lipids [64]. This could be due to the essential role cholesterol plays in maintaining neuronal function and protecting against neurodegeneration. Some studies indicate that high levels of total cholesterol and low-density lipoprotein cholesterol (LDL-C) later in life can be linked to a slower rate of cognitive decline, as these lipids are vital for maintaining cellular integrity, especially in the brain [65,66]. This is in line with our data—when adjusted by age, patients with arterial hypertension and lipid disorders were significantly younger than participants without such a condition. There is nothing in the literature that is contrary to our findings that patients with past or current stroke have lower results in cognitive tests [67,68]; the same applies to patients with HF [69]. Heart failure can negatively impact cognitive function due to its association with reduced cerebral perfusion, oxidative stress, and inflammatory processes. Studies have consistently shown that heart failure contributes to structural brain changes, such as reduced gray matter and white matter integrity, leading to cognitive decline. This aligns with findings that cardiovascular diseases, including heart failure, are significant risk factors for cognitive impairment and dementia [70]. CDT scores in patients with AF and CKD were significantly lower, but no significant difference was observed in the MMSE in those patients. This might suggest greater sensitivity of the CDT in assessing cognitive impairment in these groups.

One notable strength of this study is its emphasis on an age-homogeneous group of hospitalized seniors. Moreover, the study group is significantly large. However, there are several limitations to consider. The study is limited to older adults from central Poland who are hospitalized in a geriatric ward, have multiple medical conditions, and taking various medications. Results may differ for researchers in other regions or in younger populations. Additionally, as a cross-sectional observational study, caution is required when extrapolating the findings. A future multicenter and prospective study is necessary to address these limitations and to improve the generalizability of the results. The limitations of the cognitive assessment tools used, specifically the MMSE and the CDT, should be acknowledged. While both provide useful screening insights, their narrow scoring ranges can lead to oversimplified interpretations and limit the clinical implications. Many observed differences in the cognitive tests were not clinically significant, and these tools may lack sensitivity in detecting subtle cognitive changes, particularly across diverse educational and cultural backgrounds. Therefore, we advise caution in drawing strong clinical conclusions based solely on these scores. Future studies should incorporate more comprehensive neuropsychological evaluations to better explore the relationship between electrolyte imbalances and cognitive function.

## 5. Conclusions

Our study highlights the interplay of several factors—aging, BMI, and magnesium and calcium deficiencies—and their impact on cognitive functioning. Age and BMI are fundamental elements associated with cognitive performance. Within the studied population, the presence of HF was negatively correlated with cognitive status. Hypomagnesemia and hypocalcemia appear to independently and adversely affect the cognitive performance of older individuals, with a potential additive effect when both deficiencies co-occur. These findings underscore the necessity of monitoring nutritional levels and ion concentrations in advanced-age patients to prevent cognitive deterioration.

## Figures and Tables

**Table 1:** Comparison of basic information, cognition, and concomitant disease prevalence among the four tested groups.

Parameter	Group I Normomagnesemia and Normocalcemia (*n* = 678)	Group II Hypomagnesemia (*n* = 331)	Group III Hypocalcemia (*n* = 103)	Group IV Hypomagnesemia and Hypocalcemia (*n* = 108)	*p*-Value
Age (years), mean ± SD Median (lower; upper quartiles)	81.14 ± 7.68 82(76; 87)	81.51 ± 7.31 83 (77; 87)	83.94 ± 8.51 86 (79; 90) *^,#^	83.07 ± 8.46 84 (75; 90)	0.003 ^KW^
Sex, raw numbers (%)	Women 486 (71.6%) Men 192 (28.4%)	Women 251 (75.8%) Men 80 (24.2%)	Women 65 (63.1%) Men 38 (36.9%)	Women 74 (68.5%) Men 34 (31.5%)	0.06 ^chi2^
Body mass (kg), mean ± SD Median (lower; upper quartiles)	69.31 ± 16.14 69 (57; 80)	68.05 ± 15.65 66 (57; 77)	64.34 ± 14.53 60 (52; 73) ^#^	65.25 ± 14.98 64 (55; 75)	0.03 ^KW^
BMI (kg/m^2^), mean ± SD Median (lower; upper quartiles)	26.53 ± 5.30 25 (23; 30)	27.12 ± 5.49 27 (23; 31)	25.1 ± 4.9 24 (21; 28) ^#^	25.24 ± 4.87 24 (22; 27) ^#^	0.001 ^KW^
MMSE (points), mean ± SD Median (lower; upper quartiles)	23.46 ± 5.88 25 (20; 28)	22.65 ± 6.23 24 (19; 28)	18.66 ± 8.33 20 (14; 25) *^,#^	20.40 ± 7.55 22 (16; 26) *	0.0001 ^KW^
CDT (points), mean ± SD Median (lower; upper quartiles)	5.64 ± 3.77 6 (2; 10)	5.24 ± 3.75 5 (1; 10)	4.09 ± 3.69 4 (0; 7) *	3.98 ± 3.83 3.5 (0; 8) *^,#^	0.0001 ^KW^
Number of administered medications, mean ± SD Median (lower; upper quartiles)	4.85 ± 2.42 5 (3;6)	5.85 ± 2.45 6 (4;7) *	3.98 ± 2.47 4.(2;6) *^,#^	5.03 ± 2.39 5 (3;7) ^†^	0.001 ^KW^
Arterial hypertension, raw numbers (%)	570 (84.1%)	286 (86.4%)	82 (80.4%)	90 (83.3%)	0.48 ^chi2^
Diabetes mellitus, raw numbers (%)	181 (26.8%)	160 (48.3%) *	26 (25.2%) ^#^	35 (32.4%)	<0.0001 ^chi2^
Lipid disorders, raw numbers (%)	352 (51.9%)	147 (44.4%)	32 (31.1%) *	24 (22.2%) *	<0.0001 ^chi2^
Stroke, raw numbers (%)	100 (14.8%)	72 (21.8%) *	14 (13.6%) ^#^	18 (16.7%)	0.03 ^chi2^
Coronary artery disease, raw numbers (%)	233 (34.4%)	141 (42.6%)	31 (30.1%)	31 (28.7%) ^#^	0.01 ^chi2^
Myocardial infarction, raw numbers (%)	74 (10.9%)	31 (9.4%)	16 (15.5%)	11 (10.9%)	0.36 ^chi2^
Atrial fibrillation, raw numbers (%)	146 (21.5%)	99 (29.9%) *	28 (27.2%)	33 (30.6%) *	0.01 ^chi2^
Heart failure, raw numbers (%)	342 (50.4%)	196 (59.2%)	67 (65.1%) *	79 (73.2%) *^,#^	<0.0001 ^chi2^
Chronic kidney disease, raw numbers (%)	328 (48.4%)	182 (55.2%)	61 (59.2%)	72 (66.7%) *	0.001 ^chi2^

* Significantly different from Group I; ^#^ significantly different from Group II; ^†^ significantly different from Group III; BMI—body mass index; MMSE—Mini-Mental State Examination; CDT—Clock-Drawing Test; SD—standard deviation; ^chi2^—chi-square test; ^KW^—Kruskal–Wallis test.

**Table 2:** MMSE and CDT values according to the presence of a particular disease.

Disease	Without a Particular Disease Mean ± SD, Median (Lower; Upper Quartiles)	With a Particular Disease Mean ± SD, Median (Lower; Upper Quartiles)	*p*-Value ^U^
MMSE			
Arterial hypertension	21.4 ± 7.5, 23 (17; 28)	22.8 ± 6.3, 24 (19; 28)	0.003
Diabetes mellitus	22.5 ± 6.5, 24 (19; 28)	22.8 ± 6.6, 24 (19; 28)	0.32
Lipid disorders	21.7 ± 6.6, 23 (18; 27)	23.6 ± 6.3, 25 (20; 29)	0.0001
Stroke	22.9 ± 6.5, 24.5 (19; 28)	20.9 ± 6.7, 22 (18; 26)	<0.0001
Coronary artery disease	22.6 ± 6.6, 24 (19; 28)	22.4 ± 6.5, 24 (19; 27)	0.56
Myocardial infarction	22.6 ± 6.5, 24 (19; 28)	22.5 ± 6.5, 24 (19; 28)	0.86
Atrial fibrillation	22.7 ± 6.6, 24 (19; 28)	22.2 ± 6.4, 23 (19; 27)	0.23
Heart failure	23.6 ± 6.3, 25 (20; 28)	21.8 ± 6.7, 23 (18; 27)	<0.0001
Chronic kidney disease	22.8 ± 6.8, 25 (19; 28)	22.4 ± 6.3, 24 (19; 27)	0.19
CDT			
Arterial hypertension	5.0 ± 4.1, 5 (10; 9)	5.4 ± 3.8, 5 (2; 9)	0.27
Diabetes mellitus	5.2 ± 3.8, 5 (1; 9)	5.6 ± 3.8, 6 (2; 10)	0.10
Lipid disorders	4.9 ± 3.8, 5 (0; 9)	5.8 ± 3.8, 7 (2; 10)	<0.0001
Stroke	5.4 ± 3.8, 5 (1; 9)	5.1 ± 3.8, 5 (1; 9)	0.23
Coronary artery disease	5.5 ± 3.8, 6 (2; 10)	5.1 ± 3.8, 5 (1; 9)	0.10
Myocardial infarction	5.3 ± 3.8, 5 (1.5; 9)	5.1 ± 3.9, 5 (0; 10)	0.56
Atrial fibrillation	5.5 ± 3.9, 6 (2; 10)	4.9 ± 3.7, 5 (1; 9)	0.01
Heart failure	5.7 ± 3.9, 7 (2; 10)	5 ± 3.7, 5 (1; 9)	0.002
Chronic kidney disease	5.5 ± 3.9, 6 (1; 10)	5.1 ± 3.7, 5 (1; 9)	0.01

MMSE—Mini-Mental State Examination; CDT—Clock-Drawing Test; SD—standard deviation; ^U^ Mann–Whitney test.

**Table 3:** General linear models from multivariable linear regression for the MMSE and CDT.

	Equation	R^2^	*p*-Value
MMSE =	33.95 − (0.21 × Age) + (0.18 × BMI) + 1.69 if normal Mg and Ca; + 0.94 if low Mg; − 2.12 if low Ca; − 0.51 if low Mg/Ca; − 0.46 if present chronic heart failure *or* + 0.46 if absent chronic heart failure	0.16	<0.0001
CDT =	13.26 − (0.13 × Age) + (0.09 × BMI) + 0.68 if normal Mg and Ca; + 0.28 if low Mg; − 0.36 if low Ca; − 0.60 if low Mg/Ca	0.12	<0.0001

BMI—body mass index, MMSE—Mini-Mental State Examination, CDT—Clock-Drawing Test.

## Data Availability

The statistical data used to support presented findings may be obtained by sending a request to the corresponding author.
